# Decoding Human Personality Through Dermatoglyphics

**DOI:** 10.7759/cureus.30445

**Published:** 2022-10-18

**Authors:** Shreya Venurkar, Tripti Srivastava, Samarth Shukla, Sourya Acharya, Souradip Saha, Vaishnavi Deshpande

**Affiliations:** 1 Department of Psychiatry and Behavioral Sciences, Jawaharlal Nehru Medical College, Datta Meghe Institute of Medical Sciences (Deemed to be university), Wardha, IND; 2 Department of Physiology, Jawaharlal Nehru Medical College, Datta Meghe Institute of Medical Sciences (Deemed to be university), Wardha, IND; 3 Department of Pathology, Jawaharlal Nehru Medical College, Datta Meghe Institute of Medical Sciences (Deemed to be university), Wardha, IND; 4 Department of Medicine, Jawaharlal Nehru Medical College, Datta Meghe Institute of Medical Sciences (Deemed to be university), Wardha, IND; 5 Department of Anaesthesiology, Medical College and Hospital, Kolkata, IND; 6 Department of General Surgery, Jawaharlal Nehru Medical College, Datta Meghe Institute of Medical Sciences (Deemed to be University), Wardha, IND

**Keywords:** myers-briggs type indicator, personality types, neris type explorer, human personalities, fingerprints, dermatoglyphics

## Abstract

Background

Dermatoglyphics refers to the study of epidermal ridges or patterns on fingers (fingerprints) and palms (palmprints). These epidermal ridges grow concurrently with a fetus's neural development during the intrauterine stage of life. Determining genetic anomalies using dermatoglyphics can help identify congenital deformities, various other medical conditions, and how the brain functions well ahead of time. A self-report questionnaire called the Myers-Briggs Type Indicator (MBTI) is used to identify several psychological traits influencing how people perceive their surroundings and make decisions.

Further investigation of this connection between Dermatoglyphics and Myers-Briggs Personality types can provide helpful insight into understanding a child's potential and perception of the world at a very tender age, student's potential towards a particular profession, and guiding their career choices. Understanding a child's personality type can give the parents an edge over understanding and catering to their emotional and social needs, hiring qualified employees, etc.

Objective

The study aims to clarify the connection between Myers-Briggs personality types by utilizing fingerprint patterns.

Methodology

An analytical cross-sectional study was conducted on 200 consenting students aged 25-30 years in a rural Medical School in the backdrop of central India. The study duration was two months, and the data collection was based on NERIS Type Explorer (based on the Myers-Briggs 16 personality type). The study involved the collection of fingerprints by using ink (type) and paper. At the same time, the data analysis was done statistically using the chi-square test.

Result

Statistically, a significant association was found between a few of the personality types and fingerprint patterns using the chi-square test (P=0.05, Significant) for INTJ with whorl, INTP with whorl (two cores), INFJ with ulnar loop, ENFJ with ulnar loop, ENFP with ulnar loop, ESTJ with ulnar loop, ISTP with ulnar loop, ISFP with ulnar loop and ESTP with composite. Additionally, more than 90% of the subjects were satisfied with the accuracy of the results of the Questionnaire (Survey questionnaire by NERIS Type Explorer).

Conclusion

The study found that participants with the left loop/right loop fingerprint type made up the majority and had more moderate personality qualities. Left loop's overall average was the highest of the 16 personality types, showing that people with this fingerprint type typically exhibited apparent personality features. The 16 personality components' total average for arch/whorl was second highest, specifically in the constructs of “socially harmonious method of operation,” “strong sense of responsibility,” “enthusiastic attitude,” and "concern for others' well-being.” The total average of the arch/whorl fingerprint type was higher than the S-type/right loop fingerprint type in these four constructs, demonstrating that participants with this fingerprint type showed good leadership abilities. INFJ personality type seems to be the most occurrent among the studied population, as the study was conducted with medical school students.

## Introduction

Dermatoglyphics refers to the study of epidermal ridges or patterns present on fingers (fingerprints) and palms (palm prints) [1]. These epidermal ridges grow concurrently with a fetus's neural development during the intrauterine stage of life. Determining genetic anomalies using dermatoglyphics can help identify congenital deformities, various other medical conditions, and how the brain works [2]. Even identical twins have different fingerprints; everyone does. Unless they are harmed by dermal trauma, these fingerprints remain unchanged throughout life from the time of their creation. The fingerprints, toe prints, palm prints, and foot patterns on our ten fingers, ten toes, palmar, and plantar surfaces are reflections of the various brain regions. These dermatoglyphics can be used in the dermatoglyphics cognitive intelligence test (DMIT), which is currently used all over the world [3]. A self-report questionnaire, the Myers-Briggs type indicator (MBTI) is used to identify several psychological traits that influence how people perceive their surroundings and make decisions [4].

The indicator's goal is to use C. Jung's type theory. The core tenet of the hypothesis holds that despite seeming quite random, human behavior is highly organized and regular and is caused by specific fundamental differences in how people like to employ perception and judgment. Here, “perception” refers to the processes involved in becoming aware of objects, persons, events, or thoughts. The procedures of concluding what has been viewed are included in what is meant by “judgment.” People may exhibit equivalent variances in their interests, values, wants, and motivations due to systematic differences in what they see and the conclusions they draw. They may also differ in what they do best and what they enjoy doing the most. People may exhibit equivalent variances in their interests, values, wants, and motivations due to systematic differences in what they see and the conclusions they draw. They may also differ in what they do best and what they enjoy doing the most. The indicator adopts this working theory and makes an effort to determine people's core preferences for perception and judgment from self-reporting quickly observed reactions to investigate and consider how your preferences interact with one another in your daily life [5].

Studying the relationship between dermatoglyphics and Myers-Briggs personality types can also help students make informed job decisions by revealing how well-suited they may be for a given field. The basic questionnaire test, which offers characteristics for those choices, serves as the basis for the indicator for type measurement. There are no right or wrong responses, something that is frequently emphasized throughout the lesson. This makes it more difficult for someone being tested to “pass the test right.” The results show which option is most prevalent in each category. The two mental types of processing and orientation might be used to categorize these four preferences. Energy direction and interaction with life are both parts of exposure. Processing has to do with decision-making and information processing. Each kind describes a person's behavior and approach to reality. Additionally, it spreads into learning environments. According to experimental findings, most subjects' left/right thumbprints were left loop/right loop. The moderate personalities of persons with this fingerprint type were consistent across all 16 personality traits [6].

The study of personality characteristics, both theoretically and practically, is a crucial area of study in contemporary research fields. The accurate prediction of personality types is an area of human study that urgently needs to be developed and also has significant application in situations like hiring qualified employees or schools picking prepared pupils. A distinctive biological trait of every human being is their fingerprint. This study aims to provide a technique for determining Myers-Briggs personality types by utilizing fingerprint type. The overall finger ridge patterns on some or all of them were based on the presence or absence of circular patterns in early classification schemes that relied only on friction ridge patterns. Additionally, more samples must be produced for research in this area to confirm the known findings and make discoveries.

Indicator of Myers-Briggs type

The MBTI is one of the most widely used tools for identifying personality types. It illustrates how temperament influences actual human behavior and is based on Carl Jung's writings. The fourth dimension, related to how we interact with the environment, has been included. Based on these criteria, the Myers-Briggs model characterizes mental functioning as follows [7,8]: a perspective on the outside world - the flow of energy, interpreting (processing) data, making judgments (referred to as “judging” in Jung's work), knowing how the knowledge that represents dichotomies on bipolar scales converted into meaningful output, structuring life. They make up the opposing preferences per the given combinations. The following letter combinations are extraversion - introversion, sensing - intuition, thinking - feeling, and judging - perception: E, I, S, N, T, and J, respectively. All four elements are combined to form a personality type. As a result, the collection of preferred states represents 16 types of selected functions (table 1). The developers of the MBTI theory claim that hand use and personality type preferences are related. Humans utilize both, but one is more convenient and effective than the other. There are two crucial aspects of fingerprints: first, they do not change over time, and second, each person has a distinct fingerprint [9]. Because of the two qualities mentioned above, fingerprints have long been used for identification [10,11].

Fingerprint classification

The only criteria used in early classification schemes were friction ridge patterns and the overall ridge patterns of many or all fingers, including the presence or absence of circular patterns. The Henry Classification System was developed in India and implemented in most English-speaking countries [12]. Three fundamental fingerprint patterns-loop, whorl, and arch-make up the Henry Classification System and account for 60%-65%, 30%-35%, and 5% of all fingerprints, respectively. Other more intricate classification schemes divide patterns into loops, plain arches, or tented arches. Depending on which side of the hand the tail points, the coils may be radial or ulnar. Ulnar loops begin on the finger's pinky side, closest to the ulna, the lower arm bone. The side of the finger most comparable to the radius, the thumb side, is where radial loops begin. Plain whorls, accidental whorls, double loop whorls, peacock's eye, composite, and central pocket loop whorls are only a few sub-group classifications for whorls [12,13]. The number of different types of human fingerprints is currently unknown; however, most studies use the five main categories proposed by Henry: plain arch, tented arch, radial loop, ulnar loop, and whorl [14].

## Materials and methods

Materials

Fingerprints were taken on paper with ink. Questionnaire-based (Survey questionnaire by NERIS Explorer) study was conducted [15]. This personality assay recorded the personality types of 200 subjects. The personality types were classified and studied based on the 16 personality types by Myers Briggs. The duration of the study was two months. The test instruments used were a survey questionnaire- by NERIS Explorer (based on the Myers-Briggs 16 personality type). The fingerprint collection was done using pages, stamp ink, a scanner, and a computer/laptop.

Ethical Clearance

The Institutional Ethics Committee, in its meeting held on March 30, 2019, has approved the following research work proposed to be carried out at Jawaharlal Nehru Medical College and Acharya Vinoba Bhave Rural Hospital, Sawangi (Meghe), Wardha. This approval has been granted on the assumption that the proposed research work will be carried out in accordance with the ethical guidelines prescribed by Central Ethics Committee on Human Research (CECHR).

Methodology

It was an analytical cross-sectional study conducted over a two-month study period between August 2019 and October 2019. It was conducted on 200 consenting individuals, aged 20 to 30 years, in a rural medical school in Central India. The sample selection is done by simple random sampling giving equal probability for selection to each sample. Sampling size was calculated by using a formula n≥ Z(1−α/2) ^2​×p (1−p)​/d^2 at a 5% error margin considering prevalence (%) value of (ISTJ) 26 out of 202 samples 12.871% at priori with reference [16].

The inclusion criteria included subjects within the age group of 25-30 years, within the study setting, with fingerprint patterns that were clear and included in the scope of the study, satisfied with the accuracy of the results of the questionnaire (Survey questionnaire by NERIS Type Explorer) and who were ready to give written informed consent for the study. The exclusion criteria included the subjects who were below 20 and above 30 years, residing beyond the scope of the setting, with unclear fingerprints, which were beyond the scope of this study, not satisfied with the accuracy of the results of the questionnaire, and who were not willing to give written informed consent for the study.

Introducing the essential background of the research to the students - taking consent about their participation and use of the data in the study - taking fingerprints of the sample population- analyzing the fingerprints-conducting questionnaire-based survey for the personality type identification of each individual-identifying the personality type - correlating the personality type and the fingerprints of all the individuals - analyzing the correlation-conclusion. The data were analyzed for correlation by applying parametric tests using Software SPSS v27.0 as a part of the statistical analysis. The association between personality types and fingerprint patterns by using the chi-square test was studied. P-value <0.05 was considered significant. Additionally, subjects' satisfaction with the accuracy (over 90%) of the results of the questionnaire (Survey questionnaire by NERIS Explorer) for their personality types was important. The following parameters of fingerprints were studied and analyzed: (a) ulnar loops, (b) radial loops, (c) double loops, (d) whorls, (e) whorls (two cores), (f) arch, (g) composite.

## Results

**Figure 1:**
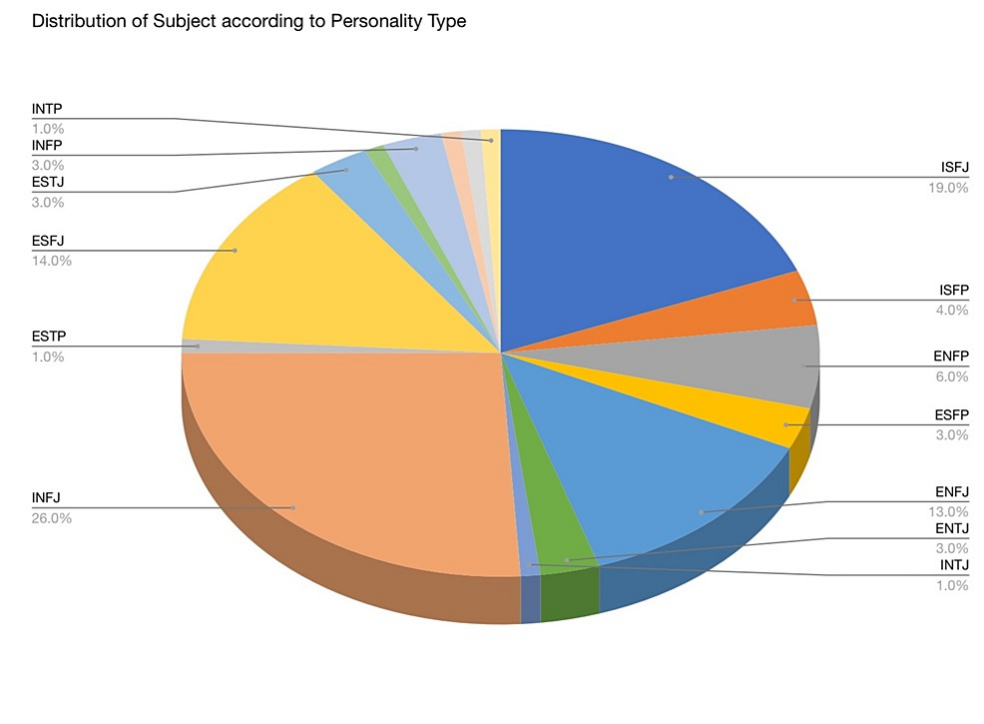
Distribution of the subjects according to personality type

In the study conducted among 200 subjects for personality types, presented in the form of a pie chart in Figure 1, INFJ accounts for 26% of all observed personality types, making it the most prevalent. In other research investigations, this personality type is not the most pervasive. In contrast, we may deduce that persons with the INFJ personality type are more likely to have their careers aligned with the medical field as the subject group comprises medical students. After INFJ, ISFJ is the study's most commonly observed personality type, with 19% of the total subject. As a result, we can infer that most of the subject population, i.e., greater than 50%, is slightly more towards introversion behavior of personality trait. ENFJ and ESFJ are 13 and 14% of the total population, respectively, and consist of the maximum population of the extroversion personality trait. The personality types with the minor population count are INTP, ENTP, ISTJ, ISTP, and ESTP. For every Myers-Briggs personality type, the Survey questionnaire by NERIS Type Explorer gives the results in the form of different names for a kind, as mentioned in Table 1 along with the percentage of the type in the subject population [15].

**Table 1:** Subject count of various personality types

Personality Type (Neris)	Personality Type (Myers-Briggs)	N	%
Architect	INTJ	2	1
Logician	INTP	2	1
Commander	ENTJ	6	3
Debater	ENTP	2	1
Advocate	INFJ	52	26
Mediator	INFP	6	3
Protagonist	ENFJ	26	13
Campaigner	ENFP	12	6
Logistician	ISTJ	2	1
Defender	ISFJ	38	19
Executive	ESTJ	6	3
Consul	ESFJ	28	14
Virtuoso	ISTP	2	1
Adventurer	ISFP	8	4
Entrepreneur	ESTP	2	1
Entertainer	ESFP	6	3

When we look at the Fingerprint Analysis, the seven categories most commonly seen in the studied population are as follows: Right loop, left loop, double-loop, whorl (plain whorl), whorl (flat whorl/whorl with two cores), composite (accidental whorl) and arch according to their order of count in the total population. The fingerprints of the two thumbs (right and left) were taken on paper with ink. 

**Figure 2:**
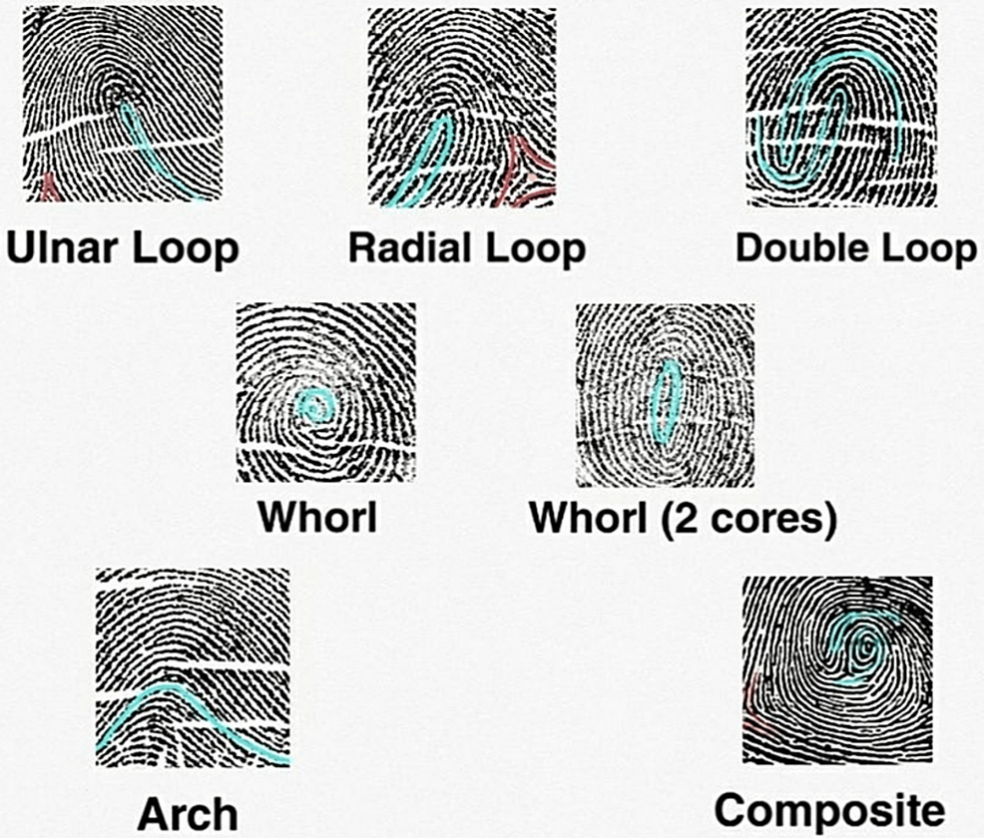
Types of fingerprints observed in the sample population

**Figure 3:**
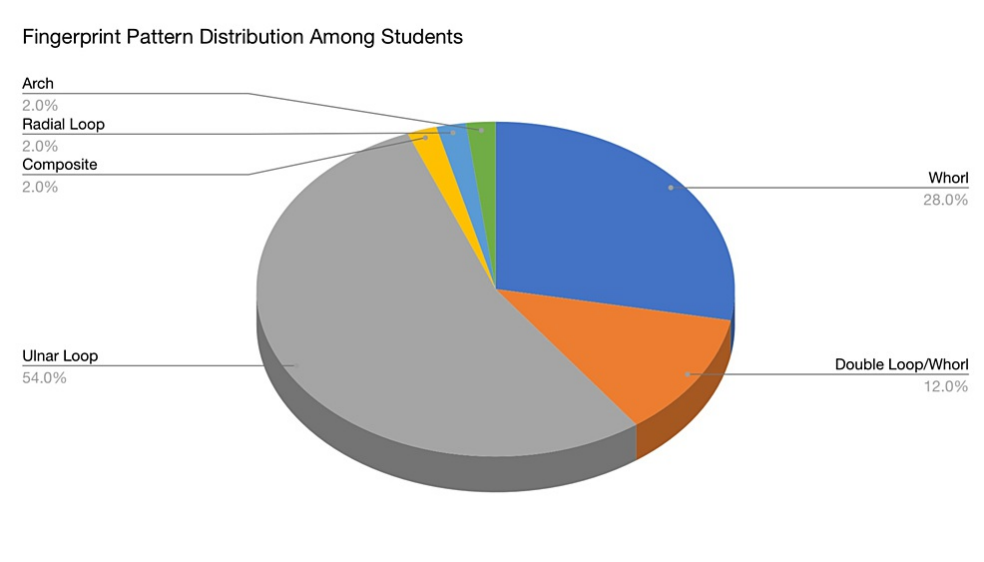
Fingerprint pattern distribution among students

**Figure 4:**
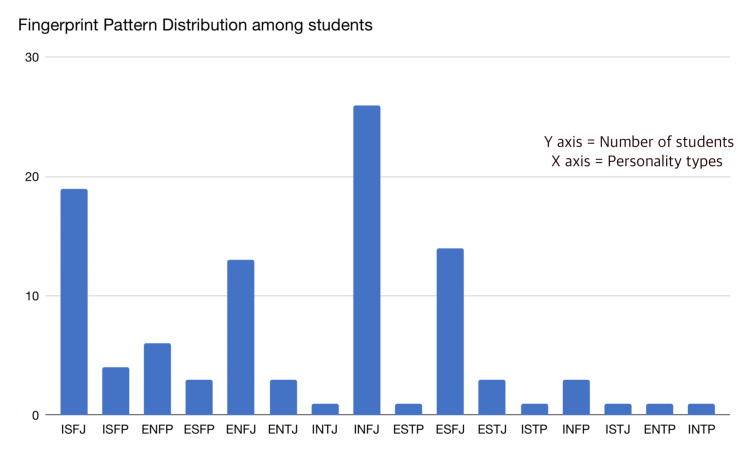
Fingerprint pattern distribution among students

Various types of fingerprint patterns are seen in Figure 2. According to the thumbprint count, Figure 3 presents the pie chart based on the observed data from Figure 4, which states that the loop form of fingerprint covers the maximum % of the type of fingerprint in the sample population. Subtypes of the loop type of fingerprint are* *radial loop, ulnar loop, and double loop. As seen in Figure 3, ulnar loop (54%) is the most occurrent fingerprint type in the loop subtypes. A double loop is a form that is almost a whorl but is not merged enough to form a complete whorl and hence is seen as a double loop. The radial loop is the least common (0%) and is restricted to only one personality type in the given sample population. But as the subject population for this type is the bare minimum, the assumption about the association of this form of a fingerprint to the personality type can have an error. The second most common personality type is the whorl. Subtypes of whorl type of fingerprints are* *plain whorl, whorl with two cores, pocket whorl, etc. Arch is one of the rarest forms of fingerprint type, followed by composite.

Association of personality types with fingerprints

According to the observed data given in Table 2, statistically, a significant association was found between a few of the personality types and fingerprint patterns using the chi-square test (P=0.05, Significant) for INTJ with whorl, INTP with whorl (two cores), INFJ with ulnar loop, ENFJ with ulnar loop, ENFP with ulnar loop, ESTJ with ulnar loop, ISTP with ulnar loop, ISFP with ulnar loop and ESTP with composite. Additionally, more than 90% of the subjects were satisfied with the accuracy of the results of the questionnaire [16].

**Table 2:** Association of personality types with fingerprints

Personality Type (Myers-Briggs)	Personality Type (Neris)	Loop			Whorl		Arch	Composite	Total	P-value
		Ulnar loop	Radial loop	Double loop	Whorl	Whorl (2 core)				
Architect	INTJ	0	0	0	2	0	0	0	2	0.0093
Logician	INTP	0	0	0	0	2	0	0	2	<0.01
Commander	ENTJ	3	0	1	2	0	0	0	6	0.775
Debater	ENTP	2	0	0	0	0	0	0	2	0.986
Advocate	INFJ	31	4	8	7	1	1	0	52	0.0073
Mediator	INFP	2	0	2	2	0	0	0	6	0.2584
Protagonist	ENFJ	18	0	0	7	1	0	0	26	0.0828
Campaigner	ENFP	6	0	5	0	1	0	0	12	0.0023
Logistician	ISTJ	2	0	0	0	0	0	0	2	0.1986
Defender	ISFJ	19	0	4	13	1	1	0	38	0.64264
Executive	ESTJ	6	0	0	0	0	0	0	6	0.0248
Consul	ESFJ	15	0	1	9	1	1	1	28	0.5876
Virtuoso	ISTP	1	0	0	0	0	0	1	2	<0.01
Adventurer	ISFP	4	0	1	1	0	2	0	8	<0.01
Entrepreneur	ESTP	0	0	0	0	0	0	2	2	<0.01
Entertainer	ESFP	1	0	1	3	1	0	0	6	0.2488
Total		110	4	23	46	8	5	4	200	

## Discussion

Dermatoglyphics and Personality type are both influenced by genetic factors. Hence, we assess the analytical potential of the correlation between fingerprint patterns and personality types [2,12]. Numerous vital fields, including medicine, psychology, and business management, do extensive research on personality characteristics for theoretical and practical purposes [12]. Each person has personality features that come from internal processes and a consistent behavioral model that enables them to identify with that behavioral model in various contexts. Emotions, motivation, and cognition are some of the internal processes underlying personality characteristics [13]. There are two crucial aspects of fingerprints: they do not change over time, and each person has a distinct fingerprint [9]. Because of the two qualities mentioned above, fingerprints have long been used for identification [10,11]. The number of different types of human fingerprints is currently unknown; however, most studies use the five main categories proposed by Henry: Plain arch, tented arch, radial loop, ulnar loop, and whorl [14]. These dermatoglyphics can be used in the DMIT, which is currently used all over the world [3]. Numerous major disciplines do an in-depth study of personality traits for theoretical and practical objectives, including medicine, psychology, and business management [13]. The following letter combinations are extraversion - introversion, sensing - intuition, thinking - feeling, and judging - perception: E, I, S, N, T, and J, respectively. All four elements are combined to form a personality type [8]. Chinese people have been using physiognomy, palmistry, bone reading, and other techniques based on physiological aspects for hundreds of years to predict a person's fate and personality. However, the evidence for a connection between personality traits and fingerprints' individually distinctive physiological characteristics has been weak up to this point.

It is crucial to analyze and adequately infer personality features in everyday life and academic research. This study has worked on 16 personality types with a questionnaire based (NERIS Type Explorer) survey questionnaire and used fingerprint classification technology [4,16]. This study discovered the capacity to infer personality kinds from fingerprint type through results comparison. The following findings are made - validity and reliability research revealed a statistically significant correlation between different fingerprint patterns and personality types, while making sure that over 90% of participants agreed that the questionnaire's answers were accurate and reliable.

Limitation of the study

The sample selection between the divisions or groups was done by randomization process; therefore, the sample size was not equally distributed among the groups for finding the association between fingerprint patterns and personality types. The study is conducted in a limited setting and age group.

## Conclusions

When personality attributes and fingerprint type were compared, it was found that participants with the left loop/right loop fingerprint type made up the majority and had more moderate personality qualities. In other words, those with this fingerprint type did not have any particularly pronounced traits from any of the 16 personality types. Left loop's overall average was the highest of the 16 personality types, showing that people with this fingerprint type typically exhibited apparent personality features. The 16 personality components' total average for arch/whorl was second highest, specifically in the constructs of “socially harmonious method of operation,” “strong sense of responsibility,” “enthusiastic attitude,” and “concern for others’ well-being.” The total average of the arch/whorl fingerprint type was higher than the S-type/right loop fingerprint type in these four constructs, demonstrating that participants with this fingerprint type showed good leadership abilities. INFJ personality type seems to be the most occurrent among the studied population, as the study was conducted with medical school students. We can correlate the medical professionals with the INFJ personality type.
